# Motivated reasoning and scientific racism in compulsion theory of human addiction: Methodological framework to promote social justice

**DOI:** 10.1111/adb.13435

**Published:** 2024-08-26

**Authors:** Lee Hogarth

**Affiliations:** ^1^ School of Psychology University of Exeter Exeter UK

**Keywords:** epistemic violence, habit and compulsion theory, social justice frameworks

## Abstract

Heinz et al. (2024) recently criticised habit/compulsion theory of human addiction but nevertheless concluded that ‘habit formation plays a significant role in drug addiction’. To challenge this causal claim, the current article develops four further methodological criticisms, that publications supporting the habit/compulsion account of human addiction: (1) under‐report contradictory observations; (2) exaggerate the process purity of positive observations; (3) under‐emphasise the low quality of epidemiological support for a causal hypothesis; (4) recapitulate the social injustice of racial intelligence era by prematurely attributing lower task performance to drug user group membership (endophenotype) without having adequately tested social, psychological, economic and environmental inequalities. Methodological guidelines are recommended to address each concern, which should raise evidence standards, incorporate social justice and improve accuracy of estimating any specific effect of addiction history on task performance. Given that construing drug users as intellectually impaired could promote stigma and reduce their recovery potential, it is recommended that scientific discourse about habit/compulsive endophenotypes underpinning addiction is avoided until these higher evidence standards are met.

## 1 UNDER‐REPORTING CONTRADICTORY OBSERVATIONS IN HABIT/COMPULSION STUDIES

Dorothy Bishop (2019)^2^ argues that confirmation bias is universal because it serves scientists' career interests (publication/grants/promotion) but invites non‐replication, reifies false theories and diverts resources. Confirmation bias is evident in the habit/compulsion literature. Specifically, there have been 15 quasi‐experiments testing whether drug use history is associated with poorer performance on tasks which putatively detect propensity to habit^3^—the tendency to undertake action without forethought of consequences (but see section below). Of these 15 tests, 3 confirmed and 12 contradicted the prediction of habit theory of poorer task performance in participants with a drug use history. The 3 confirmatory findings are routinely cited as evidence for a habit/compulsive phenotype underpinning addiction while the 12 contradictory tests are omitted or minimised by reducing their textual prominence or by exclusively focusing on their methodological limitations.

## 2 EXAGGERATED PROCESS PURITY IN HABIT/COMPULSION STUDIES

The human laboratory tasks (outcome‐devaluation and two‐step), which putatively measure propensity to habit are fundamentally flawed because trivial reductions in motivation to engage with the task, or misunderstanding of the instructions, produces exactly the same decrement in performance as would propensity to habit.^4^, ^5^ There have been verifiable attempts to bury this fundamental flaw. In the three outcome‐devaluation studies which are routinely cited as evidence for habit theory, drug users showed both poorer performance on the task (consistent with habit theory) and reduced motivation to engage with the task indicated by less accurate explicit knowledge of task contingencies (inconsistent with habit theory). The first study omitted these troubling contingency knowledge data^6^; the second study relegated them to the supplemental materials and inexplicably omitted them from a multiple regression model which adjusted for other confounds^7^; the third study demonstrated that contingency knowledge was the only significant predictor of poorer task performance, and dependence severity explained no additional variance in task performance (*R* ^*2*^ change = 0.01).^8^ This clearly demonstrated that dependent participants' reduced motivation to engage with the task was responsible for their poorer task performance, not their endophenotypic propensity to habit (as implied by the authors). The process impurity of the tasks, combined with the motivated interpretation and reporting of data, fatally undermines the empirical basis for habit/compulsion theory of addiction.

## 3 HUMAN HABIT/COMPULSION STUDIES SATISFY THE LOWEST CRITERIA FOR CAUSAL INFERENCE IN EPIDEMIOLOGY

The assertion that ‘habit formation plays a significant role in drug addiction’^1^ is also contradicted by the fact that cross‐sectional associations between drug use history and poorer task performance provide the weakest form of epidemiological evidence supporting causal inference, as evaluated against the Bradford Hill criteria described in Table 1. Considering this evaluation, authors should emphasise that it remains unknown both what caused the poorer performance in drug users and what implications it has for their future. Implying that poorer performance is prognostic of addiction risk is a clear case of theoretical over‐reach.

**TABLE 1:** Using the Bradford Hill criteria for causal inference in epidemiology to evaluate whether cross‐sectional associations between drug use history and poorer task performance (and other relevant observations) support Heinz et al.'s^1^ causal claim that ‘habit formation plays a significant role in drug addiction’.

According to the Bradford Hill criteria, what empirical observation would be needed support the claim that ‘habit formation plays a significant role in drug addiction’?	To what extent are the criteria satisfied by cross‐sectional associations between drug use history and poorer performance on the outcome‐devaluation or two‐step task (or other data)?	Criteria met?
*Effect size*: Strong relationships between drug use history and poorer task performance would strengthen the causal claim	The association between drug use history and poorer task performance is moderate in significant studies, but tiny in null studies. A meta effect size would likely be small but is hard to calculate given inconsistent reporting of the values required for meta‐analysis.	Debatable
*Consistency*: Replicable relationships between drug use history and poorer task performance would strengthen the causal claim.	As noted in the text, associations between drug use history and poorer performance in habit/compulsion tasks have not been replicated across studies.	No
*Specificity*: A unique relationship between drug use history (after adjusting for covariates) and poorer performance on certain tasks (but not others) would strengthen the causal claim.	Drug use history is a proxy for multiple psychological, social, economic and environmental disparities that could readily explain poorer task performance (by inducing low task motivation for example), which have not been adequately adjusted for. Furthermore, drug users perform less well in a multitude of cognitive tasks (memory, language, attention, etc.), suggesting no specificity to habit/compulsion tasks. Thus, it can only be concluded that people with challenging lives perform more poorly on laboratory tasks, and no more specific conclusion is empirically supportable.	No
*Temporality*: A longitudinal relationship between drug use history and poorer task performance would strengthen the causal claim if lower task performance preceded addiction.	Relevant longitudinal studies in humans do not exist, so it should be acknowledged that the cause of drug users' poorer performance, and its implication for their future, are both unknown.	No
*Biological gradient*: An incremental curve fit between greater drug use history and poorer task performance would strengthen the causal claim.	Group contrasts and regression models are employed variably across studies, so it is unclear if there is a linear or curvilinear relationship. It has been claimed that habit/compulsion only emerges at higher levels of dependence, but this claim is more ad hoc to dismiss some failed replications than quantitative.	No
*Plausibility*: A plausible theory of how habit/compulsion could cause drug use would strengthen the causal claim.	Researchers are polarised regarding the plausibility of habit/compulsion theory depending on which observations they emphasis or diminish. Other explanatory frameworks for addiction do not appeal to habit or compulsion.	Debatable
*Coherence*: Triangulation between epidemiological associations and laboratory causal effects could strengthen the causal claim.	This issue is debatable. Habit/compulsion theories rely heavily on experimental animal data, but the generalisation of effects in caged animals to free roaming humans has been questioned. Human quasi‐experiments which associate drug use history with task performance do not demonstrate causality because drug use history is not randomised (i.e., has multiple confounds). There are human studies showing experimental drug administration or stress induction causes reduced task performance, but these effects could be due to task disengagement rather than habit/compulsion, and examination of the methods sections reveals the use of p‐hacking techniques which are known to elevate false positive rates such as non‐preregistration of analytical plans, post‐hoc participant exclusions and sub‐group analyses, arbitrary time bins etc., and there have been failed replications in this space.^9^, ^10^ Drug use history has been linked to putative habit/compulsion questionnaires but the construct validity of these questionnaires is presumed. In sum, researchers weigh this evidence depending on their a priori theoretical commitment and have become polarised despite access to the same information.	Debatable

## 4 HUMAN HABIT/COMPULSION STUDIES RECAPITULATE THE SOCIAL INJUSTICE OF RACIAL INTELLIGENCE ERA

The methodological and interpretative bias that marked scientific racists in the IQ era was their comparison of racialised versus White groups on intellectual tasks and their insistence that poorer performance in the racialised group arose from race group membership (endophenotype) rather than from inequalities between groups.^11^ The critical/historical psychologist Thomas Teo defines this biased essentialist academic discourse as ‘epistemological violence’, which ‘is a practice that is executed in empirical articles and books in psychology, when theoretical interpretations regarding empirical results implicitly or explicitly construct the Other as inferior or problematic, despite the fact that alternative interpretations, equally viable based on the data, are available’.^12^ It is argued that habit/compulsion publications meet this definition of epistemological violence by failing to adequately consider inequities when explaining drug users' performance differences.

The health equity framework (HEF) provides guidelines designed to ensure that public health researchers incorporate a social justice approach by first testing whether group differences in health outcomes can be attributed to social, psychological, economic and environmental disparities, rather than group membership (endophenotype).^13^ Helms et al. (2005)^14^ specify guidelines on how these disparities should be treated in hierarchical multiple regression models. When adapted for human experimental habit/compulsion studies, these guidelines state that in step 1 the disparities should be entered to quantify the proportion of variance they explain in task performance (to formally acknowledge disparities as the primary explanatory constructs). Then in step 2, the drug use history variable should be entered (e.g., drug user vs. non‐user, drug exposure level, dependence severity, etc.) to quantify the effect size of the additional variance explained in task performance indexed by the *R* ^2^ change value. Using this socially just method, Weiss et al.^15^ found that race explained no additional variance in IQ performance over disparities, thus collapsing the essentialist presumption of the racist IQ theorists and demonstrating the power of the HEF to produce socially just scientific epistemologies.

Human experimental habit/compulsion studies have not reached the evidence standards set by the HEF. Human outcome‐devaluation and two‐step studies with drug users have collected minimal data characterising the social, psychological, economic and environmental disparities experienced by drug users versus non‐users. Where covariates of drug use history have been reported, they have been minimised as explanatory constructs for poorer task performance in favour of the drug history variable. For example, the most cited outcome‐devaluation study claiming support for habit theory^7^ reported a hierarchical multiple regression model (in the supplemental materials) where drug use history was entered in step 1, and the few disparities/confounds collected (e.g., life stress and task engagement) entered in step 2 (the reverse of HEF), thus ensuring that drug use history could be retained as an explanatory construct for poorer task performance. Presumably, the drug group effect would have been eliminated if the reverse‐ordered model suggested by HEF had been used, collapsing the key evidence for habit theory. Consistent with this assertion, in the few studies reporting adjusted models closer to the HEF guidelines, drug use history explained no significant additional variance in task performance over the disparities/confounds.^8^, ^16^ It seems likely therefore that if the evidence standards set by the HEF were employed, the empirical basis for claiming that ‘habit formation plays a significant role in drug addiction’^1^ would be eliminated and more socially just theoretical characterisation of drug users as having been subjected to social inequities would emerge.^3^

## 5 CONCLUSIONS

Four standards of scientific practice are recommended to ensure that psychologists do not produce epistemically violent characterisation of drug users (or other marginalised groups) as possessing risky endophenotypes, until there is sufficient quality of evidence to warrant this claim.^12^

- To reduce confirmation bias exaggerating marginalised group differences, empirical studies should employ Cochrane's rapid systematic review protocol to impartially enumerate all effects and give equal textual prominence to the null effects.
- To reduce exaggerated claims of specific functional deficits in marginalised groups, researchers should acknowledge the process impurity of their measures and assume that general confounds (motivation, irritation, demand characteristics, expectation, etc.) arising from inequities probably drive the effects, until proven otherwise.
- To reduce exaggerated causal inferences, marginalised group differences should be evaluated against the Bradford Hill criteria and interpreted accordingly.
- To ensure a social justice approach is adopted and avoid prematurely attributing marginalised group difference to endophenotype, the HEF hierarchical regression model guidelines^14^ noted earlier should be employed to first test social, psychological, economic and environmental disparities as explanatory constructs, and only then estimating any unique contribution of group membership.

These guidelines will hopefully encourage psychologists to stop problematising marginalised groups to meet professional incentives and instead build a socially just epistemology that could underpin social and individual change.

## CONFLICT OF INTEREST STATEMENT

None.

## Data Availability

Data sharing not applicable to this article as no datasets were generated or analysed during the current study.
